# Melanosome diversity and convergence in the evolution of iridescent avian feathers—Implications for paleocolor reconstruction

**DOI:** 10.1111/evo.13641

**Published:** 2018-11-26

**Authors:** Klara K. Nordén, Jaeike W. Faber, Frane Babarović, Thomas L. Stubbs, Tara Selly, James D. Schiffbauer, Petra Peharec Štefanić, Gerald Mayr, Fiann M. Smithwick, Jakob Vinther

**Affiliations:** ^1^ School of Earth Sciences University of Bristol 24 Tyndall Avenue Bristol BS8 1TQ UK; ^2^ Current address: Department of Ecology and Evolutionary Biology Princeton University Princeton NJ 08544 USA; ^3^ Current address: Department of Medical Biology Academic Medical Center University of Amsterdam Meibergdreef 15 1105 AZ Amsterdam The Netherlands; ^4^ Current address: Department of Animal and Plant Sciences University of Sheffield S10 2TN Sheffield UK; ^5^ Department of Biology, Faculty of Science University of Zagreb Rooseveltov trg 6 10 000 Zagreb Croatia; ^6^ X‐ray Microanalysis Core Facility University of Missouri 101 Geological Sciences Building Columbia MO 65211 USA; ^7^ Department of Geological Sciences University of Missouri 101 Geological Sciences Building Columbia MO 65211 USA; ^8^ Senckenberg Research Institute Frankfurt Senckenberganlage 25 60325 Frankfurt Germany; ^9^ School of Biological Sciences University of Bristol 24 Tyndall Avenue Bristol BS8 1TQ UK

**Keywords:** bird coloration, convergent evolution, iridescence, melanin, paleocolor

## Abstract

Some of the most varied colors in the natural world are created by iridescent nanostructures in bird feathers, formed by layers of melanin‐containing melanosomes. The morphology of melanosomes in iridescent feathers is known to vary, but the extent of this diversity, and when it evolved, is unknown. We use scanning electron microscopy to quantify the diversity of melanosome morphology in iridescent feathers from 97 extant bird species, covering 11 orders. In addition, we assess melanosome morphology in two Eocene birds, which are the stem lineages of groups that respectively exhibit hollow and flat melanosomes today. We find that iridescent feathers contain the most varied melanosome morphologies of all types of bird coloration sampled to date. Using our extended dataset, we predict iridescence in an early Eocene trogon (cf. *Primotrogon*) but not in the early Eocene swift *Scaniacypselus*, and neither exhibit the derived melanosome morphologies seen in their modern relatives. Our findings confirm that iridescence is a labile trait that has evolved convergently in several lineages extending down to paravian theropods. The dataset provides a framework to detect iridescence with more confidence in fossil taxa based on melanosome morphology.

Iridescent coloration is responsible for some of the most striking color displays seen in birds. Produced by nanostructures in the feather barbules, iridescence creates brighter, more saturated and varied colors than any other form of bird coloration (Stoddard and Prum 2011; Maia et al. 2013), which makes them particularly efficient for intra‐ and interspecific signaling (Doucet and Meadows 2009). The simplest type of iridescence is produced by a single layer of melanin granules (melanosomes) covered by a layer of keratin, which gives rise to coherent scattering of light (thin‐film interference, Prum 2006). By modifying this basic design, birds achieve a great range and intensity of colors. The thickness of the keratin layers and the melanosome layers determine the wavelength of the reflected light, and the number of layers the amount of reflected light—that is the hue and saturation respectively of the resulting color. Iridescent plumage has been documented in at least 14 of the 32 existing bird orders (Durrer 1975; Prum 2006), but there are probably many more occurrences. The majority of iridescent bird feathers contain solid cylindrical melanosomes, similar to those of black and grey feathers, but generally having a higher aspect ratio (length to width, Li et al. 2012). However, some genera have evolved hollow and/or flat forms (Fig. 1). These derived melanosomes have so far been documented in 10 orders—Trogoniformes, Galliformes, Passeriformes, Apodiformes, Piciformes, Ciconiiformes, Coraciiformes, Gaviiformes, Gruiformes (Durrer 1975;Espinosa de los Monteros 1998; Prum 2006, Hu et al. 2018), as well as Cuculiformes (this study)—each representing an independent origin. The convergent evolution of these melanosome morphologies suggests a functional role, and it has been shown that different morphologies increase the range of colors achievable by altering the optical properties of the structure. Hollow melanosomes increase the number of interfaces for light scattering, and similarly, flat melanosomes allow the addition of more melanosome layers by permitting closer packing, which will increase saturation and brightness of the produced color (Eliason et al. 2013; Maia et al. 2013). Thus, it is not only the size of melanosomes that is important for color production (through adjusting layer spacing), but also other morphological variables such as flatness and hollowness.

**Figure 1:**
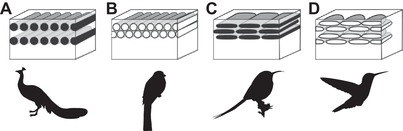
Schematic drawings of cross sectioned barbules, showing the main types of melanosome morphologies found in iridescent feathers. Silhouettes represent examples of bird families with the illustrated morphology. (A) Solid cylindrical melanosomes, (B) hollow cylindrical melanosomes, (C) flat and solid melanosomes, (D) flat and hollow melanosomes. Silhouettes: (A) Phasanidae, (B) Trogonidae, (C) Nectariniidae (sunbirds), (D) Trochilidae (hummingbirds). Image credit for silhouettes: Natasha Sinegina (A) and Katerina Ryabtseva (D) downloaded under Creative Commons license (https://creativecommons.org/licenses/by/4.0/).

The diversity of melanosome morphologies in iridescent feathers can broadly be divided into four types: solid cylindrical, solid flattened, hollow cylindrical, and hollow flattened (Durrer 1975). Previous studies have typically investigated the optics of iridescent structures in singular taxa (Yoshioka and Kinoshita 2002; Zi et al. 2003; Yin et al. 2006; Stavenga et al. 2011, 2015, 2017, 2018; Xiao et al. 2014), however, large‐scale surveys are rare. This previous work shows that melanosome dimensions and morphology can alter hue, saturation, and brightness of the color produced—but the extent of variation in melanosome morphology and its spread across avian phylogeny is little understood. Here, we quantify melanosome morphology on a large scale and test for convergence in the four types of iridescence‐generating melanosome. This offers important clues to what drives the evolution of melanosome morphologies, and in turn, the evolution of iridescence in birds.

Our second aim with this study is to improve our ability to detect iridescence in the fossil record. Melanosome morphology is distinct for brown, grey, black, and iridescent coloration, which has enabled reconstruction of colors in extinct animals where melanosomes are preserved (Li et al. 2010, 2012; Zhang et al. 2010; Carney et al. 2012). The earliest record of (feather) iridescence comes from the Late Jurassic theropod *Caihong juji*, which preserves solid flat melanosomes (Hu et al. 2018). The closely related *Microraptor* also preserves plumage predicted as iridescent, but the melanosomes are of the solid cylindrical type. Clearly, iridescence based on at least two melanosome types had already evolved by the Cretaceous, revealing a remarkable diversity of melanosome morphology early in the history of iridescent feathers. Phylogenetic bracketing, that is, inferring the trait was present in the last common ancestor of *Caihong juji* and extant birds, would extend the origin of iridescent feathers to the first paravians in the Late Middle Jurassic. It has been observed that a large expansion of melanosome morphological diversity coincided with the appearance of paravians, a transition attributed to physiology (Li et al. 2014). This also coincides with the first pinnate feathers, and potentially the first barbules. Iridescence is only generated in the barbules in living birds, therefore it is possible that the evolution of barbules, permitting iridescence to form, could equally have driven this expansion of melanosome diversity. However, the model used by Li et al. (2012) was based on a small sample of solid cylindrical melanosomes, capturing only part of the actual diversity of iridescence‐generating melanosomes. With flat melanosomes already described in the fossil record (Hu et al. 2018), and the discovery of hollow melanosomes being a real possibility, the predictive model should be based on a wide sample that encompass all the melanosome types we see in modern birds to accurately detect iridescence in the fossil record. Hu et al. (2018) expanded the data set with 32 extant species, but the sample was not phylogenetically broad (consisting mostly of hummingbirds), and because the study focused on flat melanosomes, they did not include hollow cylindrical forms. Here, we include a broader sample of iridescence‐generating melanosomes that enables us to build a more inclusive model and test the robustness of previous predictions of iridescence.

We sampled 97 extant bird species with iridescent plumage, covering all types of iridescence‐generating melanosomes and nanostructures, and quantified melanosome morphology. In addition, we analyzed two fossil birds from the early Eocene (48 million years ago) Messel fossil site in Germany, which are related to groups with derived melanosome morphologies today: cf. *Primotrogon*, a stem group representative of Trogoniformes (Mayr 2005), which have hollow melanosomes (Durrer 1975); and *Scaniacypselus*, a stem group representative of true swifts (Mayr 2003, 2015; Ksepka et al. 2013), whose sister taxon, the Hemiprocnidae, have flat melanosomes. This represents the first large‐scale study of the diversity of melanosomes in iridescent feathers.

## Material and Methods

### SAMPLING

We sampled iridescent feathers from a total of 97 bird species, covering 11 orders and 21 families (see Supporting Information Data for details of specimens), from the collections of the Zoological Museum of Copenhagen. We aimed to sample as widely as possible, both regarding phylogeny and melanosome type, and covering all four types of iridescence‐generating melanosomes (Fig. 1). Together with the dataset from Li et al. (2012), all but three of the bird orders with a documented occurrence of iridescence are represented within this study. We determined melanosome type (hollowness and flatness) following Durrer (1975). Trogoniformes and Apodiformes were more extensively sampled than other groups, as they were of particular interest for the fossil samples. For birds exhibiting more than one iridescent color, a sample was taken from each color. Feathers were plucked from skins using forceps and transferred to zip lock bags for storage and transport.

### FOSSIL SAMPLING

For fossil sampling, a sterile scalpel was used to remove small chips of feather residue averaging around 1 mm^2^. We sampled one specimen of cf. *Primotrogon* sp. from the early Oligocene of Frauenweiler, Germany (SMF Av 498, Fig. S1) and two specimens of *Scaniacypselus* from the early Eocene of Messel (SMF‐ME 599, SMF‐ME 11345 A+B, Fig. S2), from the repository at the Senckenberg Research Institute Frankfurt.

### MELANOSOME EXTRACTIONS

Melanosomes were extracted enzymatically following the method of Colleary et al. (2015), modified from Liu et al. (2003). Briefly, feather samples were washed with acetone, followed by addition of phosphate buffer (1.5 mL) and dithiohreitol (DTT, 1.5 mL), and then incubated for 24 hours at 37.5°C. We added phosphate buffer (15 mL), DTT (5 μL) and Proteinase‐K (5 mg) to the resulting pellets, and then incubated samples for an additional 24 hours. The resulting pellet was washed with water and phosphate buffer (1.5 mL), after which DTT (15 μL) and Papain (5mg) were added to each sample, and were then incubated for 24 hours. Following this, we repeated the treatment with Proteinase‐K, and then added Triton X‐100 (1.5 mL) and stirred samples for four hours. Samples were then washed, and the Proteinase‐K treatment was repeated two more times. Lastly, samples were left to dry under a laminar flow hood.

### MEASURING MELANOSOME MORPHOLOGY

Melanosome samples were coated with gold using an Edwards Scancoat six Pirani 501 sputter coater and imaged with a Zeiss Evo 15 scanning electron microscope (SEM) at the University of Bristol. The length and width of melanosomes were measured using ImageJ (Abràmoff et al. 2004), and qualitative presence and absence of flat and hollow morphologies was also assessed. Flatness could easily be determined by visual inspection of the melanosome samples under SEM (Fig. S3). The mean length, width, aspect ratio (length/width), and the coefficient of variation of length and width were calculated for each sample. We did not include skew of length and width, as has been done in previous studies (Li et al. 2012), because this will be highly sensitive to sample size. The same approach was used to measure melanosomes in fossil specimens. Our dataset was combined with that of Li et al. (2012), which includes black (*n* = 45), brown (*n* = 35), grey (*n* = 35), and iridescent (*n* = 35) colored feathers. The Li et al. (2012) data set also includes 18 samples from penguins, which were not included in our analysis. We excluded these samples because the melanosomes from penguins (including samples from black and brown feathers) have a distinct morphology unrelated to their color (Clarke et al. 2010). Penguin melanosomes are organized as clusters in the feather barbs, which is likely the reason for their specific shape. This unique structure might affect feather material properties, increasing strength to withstand the higher abrasion feathers would experience in an aquatic environment (Clarke et al. 2010). Because this variation is likely connected to the unique aquatic lifestyle of penguins, and our model is concerned with predicting color in terrestrial birds, we excluded penguin samples from the dataset.

Since sample size varied in the dataset, particularly between our data and the Li et al. (2012) data set, we conducted a series of tests to investigate whether this would affect the results of our analysis (see Supporting Information Methods and Figs. S4–S6). Briefly, using a subsampling approach, we found that for a sample size of *n* = 10, width and length measurements varied by an average coefficient of variation (CV) of 5–6%, which was considered acceptable since the average CV of length and width within each species is much higher (20%). This was not true for CV of length (a variable used in previous predictive models), which had a CV of 25% for *n* = 10 (Fig. S6C and D). Since a higher sample size cutoff would lead to substantial reductions in especially the Li et al. (2012) data set, we instead excluded length CV from our analyses. Experimenter bias was assessed by comparing measurements taken by different individuals on the same SEM image (differences ranged 3–14% on the two samples tested).

### ASSESSING HOLLOWNESS OF MELANOSOMES

We further assessed the presence or absence of melanosome hollowness using transmission electron microscopy (TEM) for extant species (in which this was not documented elsewhere, Fig. S12) and focused ion beam sectioning (FIB‐SEM) for fossil samples. Briefly, for TEM analyses, feathers were embedded in resin following the protocol from Shawkey et al. (2003), see Supporting Information Methods for details), and then cut into 70–100 nm thick cross sections with an RMC‐MT ultramicrotome 6000. Cross sections were placed on copper grids and observed with an FEI Morgagni 268D transmission electron microscope. FIB‐SEM methods follow those described in Vitek et al. (2013) and Schiffbauer and Xiao (2009, 2011), using an FEI Scios DualBeam at the University of Missouri Electron Microscopy Core Facility. FIB‐SEM milling was conducted with a Ga^+^ ion beam voltage at 10 kV. Overview SEM images following FIB‐SEM milling were collected using a Zeiss Sigma 500 VP at the University of Missouri X‐ray Microanalysis Core Facility.

### MELANOSOME MORPHOSPACE

A melanosome morphospace was created using the PCAmix method described by Chavent et al. (2014) using the R package “PCAmix.” PCAmix is a two‐step process that combines a principal component analysis (PCA) with a multiple correspondence analysis (MCA). This allows inclusion of both qualitative (flat or cylindrical and hollow or solid) and quantitative (length/width/aspect ratio) variables. We projected fossil samples into this morphospace using the “predict” function in R (R Core team 2017). To quantify morphospace occupation, we calculated the PCA convex hull volume of each melanosome category (black, brown, grey, and iridescent) using the three first PC axes. Convex hull volume is sensitive to outliers, which can inflate volume estimates (Kotrc and Knoll 2015), and we therefore also calculated alpha shape volumes. The alpha parameter regulates the radius by which empty volume is removed (as alpha approaches zero data points become isolated in space, see example in Fig. S7). The alpha values tested (1.3, 2, and 5) were chosen based on visual inspections of plotted volumes. As the sample size of iridescent colors was much larger than black, grey, and brown colors, we tested for a significant difference in morphological disparity using a randomization approach. Our null distribution was created by randomly assigning category to each sample (5000 iterations), and then calculating convex hulls/alpha shape volumes of categories for each replicate. We then calculated the average ratio between categories in the replicate data sets (null distribution) and compared it with the observed data using an exact test of goodness of fit. Another way to quantify disparity is to calculate the sum of variances statistic (SoV), which measures the spread of points in morphospace, and has been shown to be robust to sample size bias (Butler et al. 2012). We applied a bootstrap method (1000 iterations) to assess the effect of sample size and estimate 95% confidence intervals.

PCA can be misleading when the data analyzed has a phylogenetic structure (Revell 2009). We assessed the effect of phylogenetic bias on our volume estimates by comparing the volume of each category derived from a phylogenetic PCA (pPCA, Revell 2009) with a standard PCA. Since the standard PCA and PCAmix scores exhibit similar phylogenetic signal (0.54 and 0.46, respectively), using pPCA on just the continuous variables should be a good approximation of the phylogenetic bias, although the variables flatness and hollowness cannot be included.

### PHYLOMORPHOSPACE AND PHYLOGENETIC SIGNAL

To visualize phylogenetic patterns within the melanosome morphospace, we constructed a phylomorphospace. For reference phylogeny, we used the time‐scaled genus‐level tree from Cooney et al. (2017), which is a composite of the phylogenies in Prum et al. (2015) and Jetz et al. (2012). The tree was pruned to match the taxa in our data, and where species were missing, we manually added them into the tree as generic polytomies (Fig. S8). The positions of internal nodes in the phylomorphospace, that is, the inferred ancestral states between branching clades, were estimated using maximum likelihood approaches in the R package phytools (Revell 2012). Phylogenetic signal in morphospace, based on all axes of variation, was calculated using a multivariate version of Blomberg's *K* statistic (*K*_mult_, Blomberg et al. (2003); Adams (2014)) in the R package geomorph (Adams and Otárola‐Castillo 2013). *K*_mult_ has been shown to perform better for multivariate traits (Adams 2014), however, we also calculated phylogenetic signal for each principal component (PC) axis separately (Supporting Information Data). The *K* (and *K*_mult_) statistic measures the strength of phylogenetic signal relative to what is expected under a Brownian motion model of evolution, where a value of *K* = 1 represents Brownian motion, values of *K* < 1 represent data with less phylogenetic signal than predicted, while *K* values > 1 describe data with more phylogenetic signal than expected. We also quantified phylogenetic signal using Pagel's lambda (Pagel 1999), which ranges from 0 (no signal) to 1 (phenotypic variation equal to Brownian expectation). This returns similar results for individual axes (Supporting Information Data).

### TESTING FOR CONVERGENCE

We tested the strength of convergent evolution in each type of melanosome morphology using the Wheatsheaf index (Arbuckle et al. 2014). The Wheatsheaf index (*w*) is calculated by dividing the mean distance in phenotypic space for all taxa by the mean phenotypic distance for the convergent taxa (the “focal group”). The “focal group” here only included one taxon for each lineage where the specific morphology evolved. Distances are weighted by phylogenetic distance, and the index, therefore, implicitly assumes evolution according to Brownian motion. A high *w* suggests a strong signal of convergent evolution, and significance is estimated by testing the observed value against a null distribution created with a bootstrap method. The metrics was calculated for PC scores (derived from PCAmix analysis) on all five axes and standardized traits (length, width, and aspect ratio), using 1000 simulations for the significance test.

### PREDICTING COLOR IN FOSSIL FEATHERS

To predict color from melanosome morphology, we employed two commonly used predictive modeling approaches: quadratic discriminant analysis (QDA, a modification of linear discriminant analysis, which allows violation of the equal covariance assumption) and multinomial logistic regression (MLR). Quadratic discriminant analysis has been used in previous studies (Li et al. 2012; Hu et al. 2018), and using this method, therefore, allows easy comparison of our model with previous models. However, using QDA is potentially problematic as the data is not normally distributed (with variables such as aspect ratio), which is an underlying assumption of QDA. Furthermore, QDA does not allow the inclusion of binary categories, such as hollowness and flatness. Both issues can be resolved by using a logistic regression.

We selected the variables to include in the models based on Akaike information criterion (AIC) values, following the approach outlined in Grueber et al. (2011). Using the *dredge* function in the R package “MuMIn”, we constructed all possible models out of the total variables in the dataset (length, diameter, aspect ratio, hollowness, and flatness), and ranked them after AIC value. This resulted in a QDA model including the variables length, aspect ratio, and diameter; and diameter, aspect ratio, flatness, and hollowness for the MLR model.

Because qualitative variables such as hollowness and flatness cannot be included in a QDA, we tested a model both excluding and including the hollow/flat melanosome data. The latter could be used if hollowness and flatness had already been excluded based on SEM analysis. We also built a QDA model based on only the Li et al. (2012) data for comparison.

Accuracy of the models were checked using repeated *k*‐fold cross‐validation (five repeats), and significance was determined using an exact test. We calculated Cohen's Kappa, which measures the concordance of predictions, accounting for the possibility that agreement could be due to chance. Cohen's Kappa ranges from 0 to 1, where a value of 1 equals perfect agreement and Kappa = 0 indicates that the predictions are not better than expected by chance.

It has been suggested that volume reduction may occur during the fossilization process (McNamara et al. 2013; Colleary et al. 2015), and we tested how sensitive each model was to potential shrinkage in melanosome dimensions (model decay). We tested 10%, 20%, and 30% shrinkage (based on the results of the maturation study by McNamara et al. 2013), and calculated the percentage of predictions that had changed from the unaltered data to the data with 30% shrinkage. This was used as an indicator of model decay, where a high decay percentage suggests the model is very sensitive to melanosome shrinkage, and a low decay percentage suggests predictions are little affected by shrinkage.

## Results

### MELANOSOME MORPHOSPACE OCCUPATION

**Figure 2:**
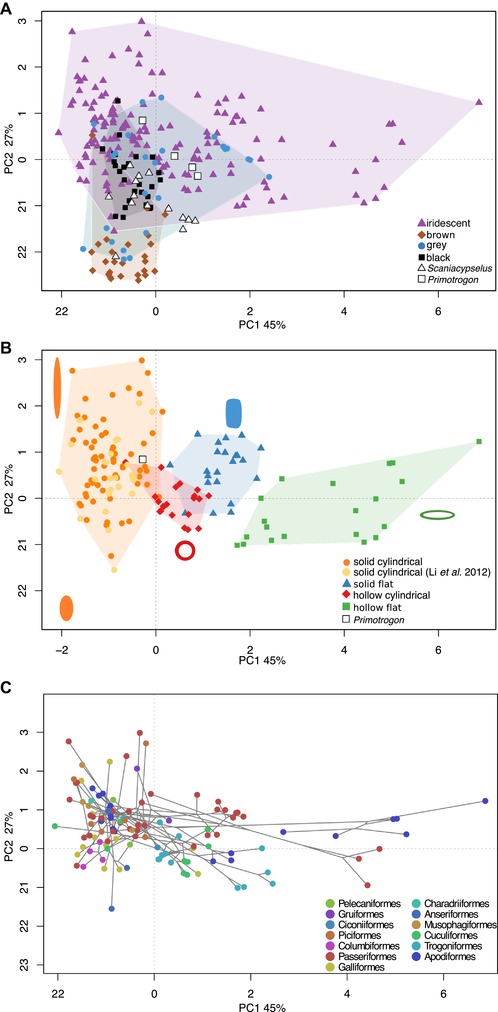
Melanosome morphospaces. PC1‐2 explains 45% and 27% of variation respectively. (A) Morphospace occupation for melanosomes from black, brown, grey, and iridescent feathers. Convex hulls are draw around each group (shaded areas). Predicted scores for fossil samples are plotted in white. (B) Morphospace occupation for iridescence‐generating melanosomes subdivided in four categories (solid cylindrical/solid flat/hollow cylindrical/hollow flat). Melanosome diagrams exemplify melanosome morphology in different areas of the plot. The fossil sample predicted as iridescent (from cf. *Primotrogon*) is plotted in white. (C) Phylomorphospace of (B) showing the spread of bird orders in melanosome morphospace.

**Figure 3:**
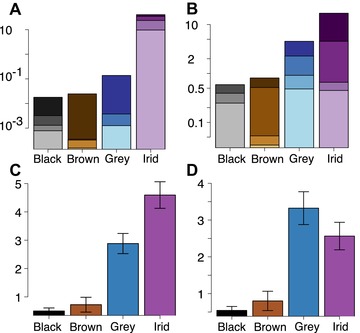
Disparity of melanosomes from black, grey, brown, and iridescent feathers. (A) Morphovolumes (based on the first three axes of variation of the PCAmix analysis), (B) Morphovolumes (based on the first three axes of variation of the pPCA analysis); (C) Sum of variances for the PCAmix analysis. (D) Sum of variances for the pPCA analysis. Darker colors in (A) and (B) indicate a higher alpha value (less volume removed), where the darkest color is the raw convex hull volume. Error bars in (C) and (D) mark the 95% confidence interval produced from the bootstrap analysis.

The variables explaining most of the variation on the first two PC axes are diameter, aspect ratio, length, and flatness (Fig. S9), and PC 1–2 together account for 72% of the variance. Flat and hollow melanosomes markedly increase the morphospace occupation of iridescence‐generating melanosomes (Fig. 2A and B) compared to the data of Li et al. (2012) who studied only solid cylindrical melanosomes. The convex hull volume of iridescence‐generating melanosomes is greater than the combined volume of melanosomes found in all other colors (Fig. 3A), and the randomization test confirms this ratio is significant compared to a null distribution (exact multinomial test, *P* < 0.001). For the lowest alpha shape value (1.3) the difference is still significant (p = 0.05), despite a dramatic drop in morphovolume. Confirming greater disparity in the iridescent category, the SoV statistic is also significantly greater for iridescence‐generating melanosomes than any other category, followed by grey (Fig. 3C).

The pPCA and standard PCA yielded nearly identical volume estimates (see Supporting Information Data for PCA volumes and Fig. S10 for plots), suggesting that volumes are not biased by phylogenetic structure in the data, and supporting the conclusion of Polly et al. (2013) that application of phylogenetic PCA to morphometric data results in a rigid rotation in space. The volumes are, as expected, lower for the iridescent category compared to the PCAmix results (Fig. 3B), as the standard PCA/pPCA exclude two important shape variables (flatness and hollowness). Interestingly, for lower alpha values, the grey category is as variable or more variable in morphology than the iridescence category. This is confirmed by the SoV analysis, in which pPCA/PCA scores result in a (nonsignificant) higher value in the grey category (Fig. 3D). Thus, accounting for phylogeny does not alter volumes or sum of variances, but excluding the hollow/flat dimension reveals that in length and diameter, the grey category is as variable as the iridescent category.

Melanosome morphology showed intermediate phylogenetic signal. A *K*_mult_ value of 0.54 implies that the magnitude of phylogenetic signal is less than predicted under Brownian motion, however, it is still significantly greater than random expectation if traits had no phylogenetic signal at all (*P *< 0.001). When hollowness and flatness are excluded, that is, when applying a standard PCA, a similar phylogenetic signal of 0.46 is recorded (*P *< 0.001). This validates that comparing volume estimates between a PCA and pPCA is an appropriate approximation of the phylogenetic bias seen in the PCAmix results.

### CONVERGENCE

**Figure 4:**
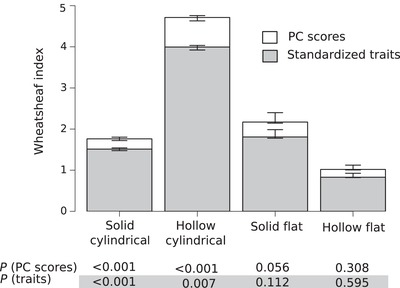
Morphological convergence in each type of melanosome in iridescence‐generating melanosomes. Convergence, as measured with the Wheatsheaf index, is shown for both PCAmix scores (white) and standardized traits (length, width, and aspect ratio, in grey). Error bars represent 95% confidence intervals from 1000 bootstrap replications, and *P* values for each measure are shown below the bar chart.

The Wheathsheaf index (*w*) confirms that iridescence‐generating melanosomes have converged in morphology within different types: significantly so in solid cylindrical and hollow cylindrical types, and close to significant in the solid flat type (Fig. 4). For those groups, it is also true that length, width, and aspect ratio have converged, even when the round/flat and hollow/solid dimensions are discounted. In these instances, the recorded *w* are lower. This is expected as convergence in the hollow and flat dimension is not included in these measures. The hollow flat type records a *w* barely greater than 1 for PCAmix scores, and this number falls below 1 when only length, width, and aspect ratio are considered. This suggests that the variation is greater within this group, than that seen among all melanosomes—although neither of these results are significant.

### MODELS AND PREDICTIONS

The multinomial logistic regression (MLR) outperforms the QDA models in all three measures of model performance: accuracy (83%), level of decay (50%), and Kappa (0.65) (Table 1). Although the QDA with all data included (total model) increased accuracy compared to the original Li et al. (2012) model, it records a lower Kappa. This is probably due to a bias toward predicting iridescence, which by chance will be more likely to be correct as the iridescent sample is larger. The QDA with only solid cylindrical melanosomes included (SC model) improved accuracy only marginally compared to the original Li et al. (2012) model.

**Table 1:** Model performance for the four predictive models tested. Model decay describes how robust model predictions are to possible melanosome shrinkage during fossilization

	Accuracy	Model decay	*P*	Cohen's Kappa
**Multinomial logistic regression**	83%	50%	<0.001	0.65
**QDA total**	73%	83%	<0.001	0.51
**QDA solid cylindrical**	66%	83%	<0.001	0.43
**QDA Li et al. (** **2012** **)**	63%	83%	<0.001	0.42

**Figure 5:**
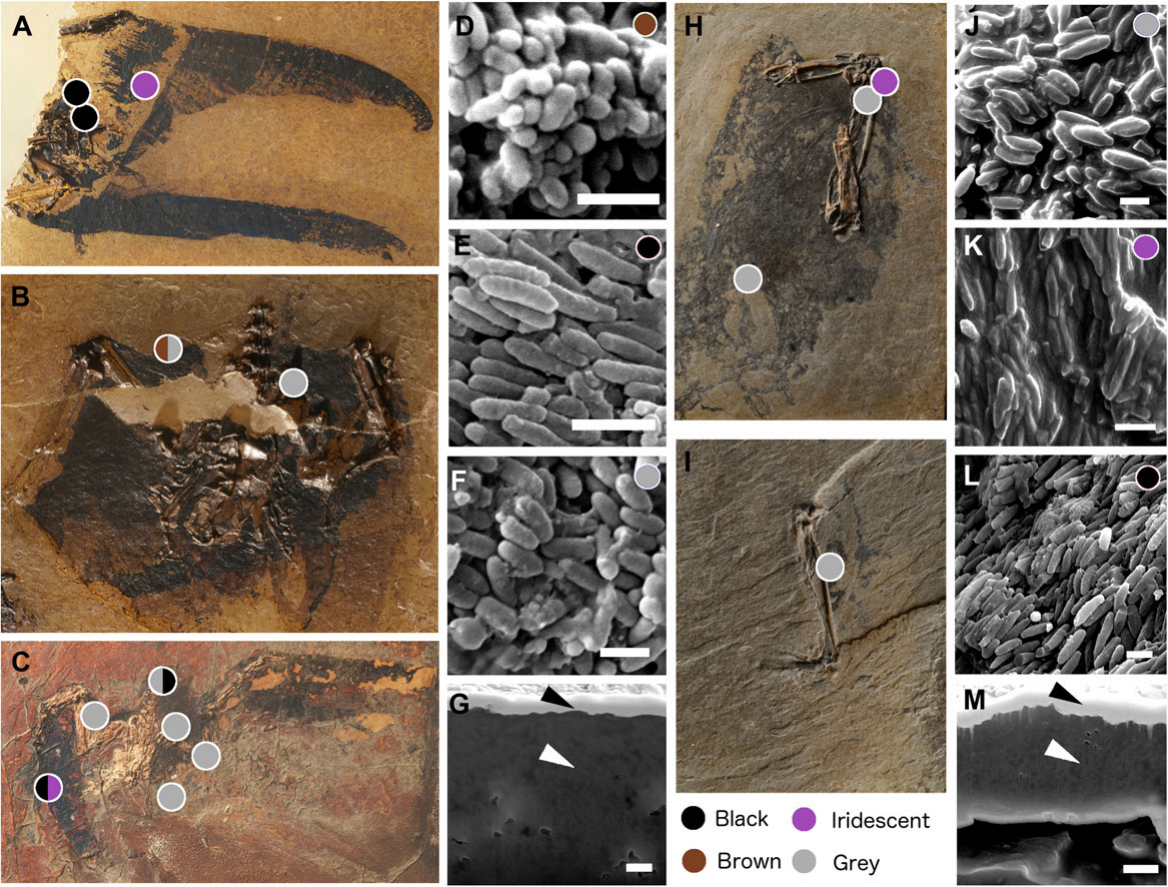
Fossil specimens and examples of fossil melanosome samples for each predicted color. Sample sites are marked by colored dots, where color indicates predicted color (details of prediction posterior probabilities can be found in Supporting Information Data). (A) *Scaniacypselus szarskii* (SMF‐ME 11345A), (B) *S. szarskii* (SMF‐ME 11345B), (C) *S. szarskii* (SMF‐ME 599), (D) morphology of fossil melanosomes in *S. szarskii* predicted as brown plumage, (E) morphology of fossil melanosomes in *S. szarskii* predicted as black plumage, (F) morphology of fossil melanosomes in *S. szarskii* predicted as grey plumage, (G) cross section of fossil sample from *S. szarskii* (SMF‐ME 599) predicted as black plumage (sample surface and cross‐sectioned area indicated with black and white arrows respectively), (H) cf. *Primotrogon* sp. (SMF Av 498a), (I) cf. *Primotrogon* sp. (SMF Av 498b), (J) morphology of fossil melanosomes in cf. *Primotrogon* sp. predicted as grey plumage, (K) morphology of fossil melanosomes in cf. *Primotrogon* sp. predicted as iridescent plumage, (L) morphology of fossil melanosomes in cf. *Primotrogon* sp. predicted as black plumage, (M) cross section of fossil sample from cf. *Primotrogon* (SMF Av 498a), predicted as iridescent plumage (sample surface and cross‐sectioned area indicated with black and white arrows respectively). Scale bars in (D–G) and (J–M) equal 1 μm.

According to the MLR model, *Scaniacypselus* is predicted as brown, black, iridescent, and grey, and cf. *Primotrogon* as grey and iridescent (Fig. 5). However, only 10 of the 18 predictions have a posterior probability equal to or over 50%, under which *Scaniacypselus* is predicted as grey and brown, and cf. *Primotrogon* grey and iridescent (see details in Supporting Information Data).

The SC model and Li et al. model show almost perfect agreement (except for one case where the QDA SC model predicts iridescent and Li et al. model black), while the total model is similar but predicts a higher number of iridescent samples.

## Discussion

### DIVERSITY AND CONVERGENCE IN MELANOSOME MORPHOLOGY

Our results demonstrate an astonishing diversity of melanosome morphologies in iridescent feathers. Partly, this is expected, as flat and hollow forms are only found in iridescent feathers, and therefore expand into areas of melanosome morphospace unoccupied by non‐iridescent forms. However, length and width are also much more variable in the iridescent category (Fig. 3B and D, with the possible exception of melanosomes in the grey category). Contrasting with this diversity is the striking convergence in morphology within different melanosome types in the iridescent category (Fig. 4). Again, this convergence is present even when qualitative characters are discounted, suggesting that the evolution of a particular melanosome type tends to favor the evolution of particular length and width dimensions. What is driving these patterns of diversification and convergence?

Uniquely to melanosomes found in feathers with structural coloration, morphology plays a direct role in the color production. Melanosome thickness is one of the parameters that can be changed in a typical multilayered iridescent structure to adjust layer spacing (the other parameters being the thickness of keratin layers, and the thickness of the keratin cortex), and thus the hue produced. In the case of two‐dimensional lattices (e.g., in peafowl), melanosome width will also affect color production through adjusting the lattice spacing in the horizontal direction. It is therefore tempting to link the diversity of morphology to the diversity of color seen in iridescent structures, which is also greater than that of any other type of coloration (Stoddard and Prum 2011). However, the length of melanosomes is effectively unimportant, as the melanosomes in the barbule functions as a layered stack or two‐dimensional lattice, not individual particles. Similarly, in one‐dimensional lattices (i.e., a simple multilayer structure with no spacing between melanosomes in the horizontal direction), melanosome width should not impact color production. One explanation for the variability in melanosome length, and melanosome width in the case of simple multilayers, could be that overall shape is important during the deposition of melanosomes in the barbule during feather development. It is speculated that melanosomes organize in the barbules through a self‐assembly mechanism via depletion‐attraction forces (Maia et al. 2012; Shawkey et al. 2015), and it is likely that melanosome morphology will affect this process as the surface tension across the melanosome surface would change. Different melanosome types (cylindrical, round, hollow, and solid) and different structures, such as single layer, multiple layer, and hexagonal packing, might have different optimal melanosome morphologies. In this way, melanosome length could indirectly affect variables that determine color production—layer thickness, spacing, and configuration.

It has been suggested that the complexity of iridescent structures in bird feathers facilitates modular color evolution, where saturation, hue, and brightness can be tuned independently using melanosome dimensions, layering, and numbers of layers (Eliason et al. 2015). Our data might reflect how this space of possible configurations has been explored by birds, by evolving a huge variability in melanosome shapes.

Given the inherent dependency of melanosome morphology in the development of iridescent nanostructures during feather growth, we might expect that melanosome type places some constraints on what dimensions are optimal for developing a particular iridescent structure. Supporting this, three of four melanosome types have converged in length, width, and aspect ratio (non‐significant for solid flat melanosomes). It is interesting to ask why these particular dimensions might be optimal for these types of melanosomes. Optical modeling, but also a greater understanding of the development of these structures in the feather, is needed to answer this question. We cannot exclude the possibility, given the enormous diversity of bird species, that more variation exists, but has not been sampled. However, it is still interesting to see this level of convergence over such great phylogenetic distances (note that also the Cretaceous theropod *Caihong juji* seems to conform to the approximate typical dimensions of modern flat melanosomes, Fig. S11).

Hollow flat melanosomes stand out from the rest by being particularly varied. In fact, they are so varied in morphology they have a *w *< 1 when flatness/hollowness is excluded—meaning that they are “more variable” in dimensions than the average of all melanosome types. Why has this type not converged?

First, it is important to note that the Wheatsheaf index only calculates the relative phenotypic distances, but does not consider position in morphospace. While it is true that hollow flat melanosomes show more shape variation than other types, it is also obvious from studying the morphospace plot that this is mainly achieved through expanding into areas of morphospace unoccupied by other groups, not through overlap. In this group, we find unusually large melanosomes with low aspect ratios (e.g. the hummingbird *Colibri coruscans* has melanosomes of a length of 2.5 μm, to compare with the average of 1 μm). The phylogenetically distant African starlings and hummingbirds have both evolved in the direction of acquiring such large, wide melanosomes—which does appear to be unique for hollow flat melanosomes. However, there are also relatively small melanosomes in this group, such as in the quetzal, and this great range of morphologies result in a low *w*.

A flat hollow morphology allows the greatest number of theoretically independent shape variables—length, width, thickness, and air cavity size can all be adjusted (cf. length and diameter (width always equals thickness) in solid cylindrical melanosomes). This allows for many more potential shapes and therefore also more potential solutions to any one problem (i.e., producing a particular color). In such a situation, we should expect less convergence. Alternatively, this great variation reflects true greater variation in color among birds with hollow flat melanosomes. This could be tested by comparing the range of colors produced by different types of melanosomes.

### COLOR PREDICTIONS—MODEL PERFORMANCE

The MLR model clearly performed best compared to the two QDA models, and has an accuracy of 83% compared to 63% for the Li et al. model. When only solid cylindrical melanosomes are included, all models perform equally (77% accuracy), hence the increased accuracy is due to the ability of the MLR model to account for a more variable sample of iridescence‐generating melanosomes, reflecting the true variation in melanosome shapes. Thus, predictions in previous analyses are likely to be robust if only solid cylindrical melanosomes are present in the sample (note that only three predictions differ between the Li et al. and MLR model for our fossil samples). However, in addition to providing a reliable method to predict color categories for any type of melanosome, the MLR model is also more robust to variations in sample size, deviations from normality, and melanosome shrinkage.

The original Li et al. model used a stepwise approach for variable inclusion, which resulted in a model with seven variables: length, diameter, aspect ratio, length CV, diameter CV, and skew of aspect ratio. While previous studies cite 82% accuracy for this model, not all variation was included in the analysis (excluding flat and hollow melanosomes that overlap with black and grey categories), and a low Cohen's Kappa suggests the accurate predictions could be partly due to chance. Stepwise methods should be avoided if possible as they are prone to overfitting, resulting in high performance on the training data, but poor performance on new data (Foster and Stine 2006; Whittingham et al. 2006). In addition, we show here that CV (and likely skew) are measures sensitive to sample size, which can be a problem when dealing with fossil samples.

How well does the MLR model perform on a fossil sample with flat and/or hollow melanosomes? The recently described theropod *Caihong juji*, with the first documented fossil flat melanosomes, presents a good test case. Using length and width measurements reported by Hu et al. (2018), the MLR model accurately predicts iridescence with 99% posterior probability, while the Li et al. model predicts grey color for the flat melanosomes with 99% posterior probability (Supporting Information Data).

A disadvantage of the MLR model might be that it returns on average much lower posterior probabilities for predictions, with several samples under the threshold value of 50%. However, although the posterior probabilities of the QDA analyses are very high, the accuracy of the model is lower than the MLR. This suggests posterior probabilities from QDA models should be interpreted with caution as they might lead to overconfidence in color predictions. Nonetheless, the failure of the MLR to categorize several of the samples suggests basic measurements such as length and width are often not enough to distinguish categories. In some cases, length and width may be the only reliable data that can be collected from fossil specimens, and in these situations, our data suggest we should not expect the probabilities of predictions to be particularly high, and sometimes inconclusive. However, where preservation is better, it should be possible to use more sophisticated methods to measure morphology, such as Fourier Elliptical Analysis. This might be a fruitful way to improve model performance further.

### FOSSIL PLUMAGE COLOR PREDICTIONS

Our results show that neither *Scaniacypselus* nor cf. *Primotrogon* exhibited the derived melanosome morphology seen in their modern lineages (Fig. 5). Since none of the samples from *Scaniacypselus* are predicted as iridescent (with >50% posterior probability), the absence of flat melanosomes could simply reflect the absence of iridescence. In contrast, cf. *Primotrogon* probably did have iridescent plumage, but utilized solid cylindrical melanosomes for this purpose, not hollow melanosomes as in all its extant relatives. The exact color produced by the feathers would depend on both the keratin and melanosomes, and since keratin generally does not survive the fossilization process (Saitta et al. 2017), hue cannot be reconstructed. Several samples were predicted as grey for both taxa, and we note that while grey is certainly a common plumage color, it is also the color of down. The condition of the fossils does not allow us to differentiate between whether the samples derive from covert feathers, or underlying down (there are no clear outlines of feather vanes, Fig. 5). We therefore refrain from drawing detailed conclusions on the plumage color pattern of *Scaniacypselus* and cf. *Primotrogon*.

Nevertheless, our finding that cf. *Primotrogon* exhibited iridescence is interesting from the perspective of melanosome evolution, as it suggests that iridescence evolved in Trogoniformes before derived melanosome morphologies (i.e., flat and/or hollow morphologies). This tentatively supports irreversible melanosome evolution suggested by Maia et al. (2013), who found shifts in African starlings between hollow, flat, and cylindrical forms, but not from derived forms to the ancestral solid and cylindrical morphologies. Similarly, Gammie (2013) found that hollow melanosomes have evolved three times in Galliformes, with no reversals to solid forms. It is interesting to note that the predicted iridescence‐generating melanosomes of cf. *Primotrogon* falls close to the morphology of modern hollow cylindrical melanosomes (Fig. 2B). Considering the high morphological convergence in the hollow cylindrical type (Fig. 4), it might be that transitions to hollowness is easier from a solid melanosome that already has similar dimensions, compared to more extreme shapes (e.g., very elongate forms). Such biases might explain the patchy distribution of hollow melanosomes in the bird tree. Studying fossil melanosomes gives us a unique opportunity to explore potential patterns of historical contingency, which could be important in understanding avian color diversity (Nordén and Price 2018).

## CONFLICT OF INTEREST

The authors declare no conflict of interest.

Associate Editor: I. Lovette

Handling Editor: P. Tiffin

## Supporting information

**Figure S1**. Sample sites and SEM images of samples for cf. *Primotrogon* sp. Samples are marked with white dots and numbered. a) *Primotrogon* sp. (SMF Av 498a). b) cf. *Primotrogon* sp. (SMF Av 498b). 1–4, corresponding SEM images to sample sites in a and b. Scale bars in 1–3 equal 2μm, scale bar in 4 equal 1μm.
**Figure S2**. Sample sites for *S. szarskii*. a) *S. szarskii* (SMF‐ME 11345B), b) *Scaniacypselus szarskii* (SMF‐ME 11345A) c) *S. szarskii* (SMF‐ME 599). Sample sites are marked with white dots and numbered. 1–14, corresponding SEM images to sample sites in a‐c. Scale bars equal 1μm in 1–2, 4 and 8, 2μm in 3 and 10–12, 4μm in 5,9 and 13, and 5μm in 14. White arrows in panel 6 and 14 points to examples of melanosomes.
**Figure S3**. Flat/cylindrical morphology could easily be assessed using SEM images. Example of melanosome sample classified as exhibiting flat morphology (left) and a sample exhibiting cylindrical morphology (right).
**Figure S4**. Diameter and length for solid cylindrical melanosomes in the Li et al. (2012) data (black) and our data (white).
**Figure S5**. Sample size distribution in the Li et al. (2012) dataset (black) and our data set (white).
**Figure S6**. Effect of sample size on morphological variables. A sample of n, where n is 1–100, was drawn 200 times from the original distribution of eight species. The resulting distribution for increasing sample size is shown for length (A), diameter (B), and coefficient of variation of length (C‐E). The solid line marks the mean of the 200 draws.
**Figure S7**. Example of the effect of changing the alpha parameter from a higher number (top) to a lower value (bottom). Areas with few data points have been “scooped out” resulting in a smaller volume for low alpha values.
**Figure S8**. Informal phylogeny used for constructing a phylomorphospace. Trimmed versions of this tree were used for calculating phylogenetic signal and convergence analysis.
**Figure S9**. Loading plot for PCAmix analysis. ld = aspect ratio.
**Figure S10**. Melanosome morphospaces for PCA and pPCA analyses. Morphospace occupation for melanosomes from black, brown, grey and iridescent feathers using PCA scores (A) and pPCA scores (C). Morphospace occupation for iridescence‐generating melanosomes subdivided in four categories (solid cylindrical/solid flat/hollow cylindrical/hollow flat) using PCA scores (B) and pPCA scores (D). Convex hulls are draw around each group (shaded areas), color code as in Fig. 2.
**Figure S11**. Morphospace position of flat melanosomes of the Cretaceous theropod *Caihong juji* (white diamond, after data given in Hu et al. 2018) in a PCA plot excluding flat/hollow dimensions. Color code as in Fig 2. Note that the length/width dimensions of *Caihong* are similar to that of modern birds with flat melanosome morphologies (blue and green points).
**Figure S12**. Cross sections of barbules of feather samples that were checked for melanosome hollowness. A) *Phaenicophaeus diardi diardi*, B) *Hirundo smithii*, C) *Tachycineta bicolor*, D) *Tauraco livingstonii*, E) *Psalidoprocne nitens*, F) *Chalcomitra senegalensis*, G) *Euphagus cyanocephalus*, H) *Galbula albirostris*, I) *Phaenicophaeus curvirostris*, J) *Lybius dubius*, K) *Psophia crepitans*, L) *Molothrus oryzivorus*, M) *Crotophaga major*, N) *Centropus sinensis*, O) *Centropus ateralbus*, P) *Centropus violaceus*, Q) *Eudynamys scolopacea*, R) *Galbula leucogastra*, S) *Surniculus lugubris*. All scale bars equal 1μm.
