# Supplementary material for: Melanosome diversity and convergence in the evolution of iridescent avian feathers—Implications for paleocolor reconstruction
Source: Evolution. 2018 Nov 26;73(1):15–27. doi: 10.1111/evo.13641 (PMC6587736; doi:10.1111/evo.13641)
Supplement: Supplementary file 2 — Figure S1. Sample sites and SEM images of samples for cf. Primotrogon sp. Samples are marked with white dots and numbered. a) Primotrogon sp. (SMF Av 498a). b) cf. Primotrogon sp. (SMF Av 498b). 1–4, corresponding SEM images to sample sites in a and b. Scale bars in 1–3 equal 2μm, scale bar in 4 equal 1μm. Figure S2. Sample sites for S. szarskii. a) S. szarskii (SMF‐ME 11345B), b) Scaniacypselus szarskii (SMF‐ME 11345A) c) S. szarskii (SMF‐ME 599). Sample sites are marked with white dots and numbered. 1–14, corresponding SEM images to sample sites in a‐c. Scale bars equal 1μm in 1–2, 4 and 8, 2μm in 3 and 10–12, 4μm in 5,9 and 13, and 5μm in 14. White arrows in panel 6 and 14 points to examples of melanosomes. Figure S3. Flat/cylindrical morphology could easily be assessed using SEM images. Example of melanosome sample classified as exhibiting flat morphology (left) and a sample exhibiting cylindrical morphology (right). Figure S4. Diameter and length for solid cylindrical melanosomes in the Li et al. (2012) data (black) and our data (white). Figure S5. Sample size distribution in the Li et al. (2012) dataset (black) and our data set (white). Figure S6. Effect of sample size on morphological variables. A sample of n, where n is 1–100, was drawn 200 times from the original distribution of eight species. The resulting distribution for increasing sample size is shown for length (A), diameter (B), and coefficient of variation of length (C‐E). The solid line marks the mean of the 200 draws. Figure S7. Example of the effect of changing the alpha parameter from a higher number (top) to a lower value (bottom). Areas with few data points have been “scooped out” resulting in a smaller volume for low alpha values. Figure S8. Informal phylogeny used for constructing a phylomorphospace. Trimmed versions of this tree were used for calculating phylogenetic signal and convergence analysis. Figure S9. Loading plot for PCAmix analysis. ld = aspect ratio. Figure S10. Melanosome morpho [file EVO-73-15-s002.docx]

**Supplementary material**

Supplementary methods

*Sample size bias*

Sample sizes are not equal in our dataset (Fig. S5), and we investigated the effect of sample size using a subsampling approach (Fig. S6). A sample of 1-100 were drawn from the original data 200 times, and standard deviation and coefficient of variation (CV) was calculated of length, diameter and length CV to estimate the relationship between sampling and these variables. We tested two species in each category of melanosome morphology (solid cylindrical, hollow cylindrical, solid flat, hollow flat). We found that at a sample size of 10, the variability in length and diameter, calculated as CV, was 5.9% and 5% respectively. This was considered acceptable, and 10 was therefore used as cut-off point for inclusion in the dataset. In contrast, length CV varied substantially when sample size was 10, with a CV of 23.6%. This is perhaps unsurprising as variation will naturally move towards the mean as a bigger sample is collected. We therefore excluded length CV, despite its usage in previous analysis. The alternative, to exclude all samples with low sample sizes, would have substantially reduced sample sizes in black, grey and brown categories.

These results are important as the sample size is substantially lower in the Li et al. (2012) datset compared to our dataset, potentially introducing systematic bias between color categories. Furthermore, Li et al. (2012) collected data using a different method than used here (they measured melanosomes from barbule cross sections). Both factors might have introduced differences. This is particularly problematic as potential bias would affect the grey, brown and black categories, but not the iridescent category (where our sample constitutes the majority). While we could not compare each category against our own data (as we only sampled iridescent feathers), both datasets overlap in the solid cylindrical iridescence-generating melanosomes. We conducted analyses of variance (ANOVA) between solid cylindrical iridescence-generating melanosomes in the Li et al. (2012) dataset and our dataset to check for such effects, finding that neither length or diameter was significantly different (Supplementary data). However, length CV was significantly greater in the Li et al. (2012) dataset (p=0.03). This suggest that the great variability in length CV estimated earlier in subsampling analyses do indeed bias the Li et al. (2012) samples compared to our dataset.

Based on these explorations, we only included length, diameter and aspect ratio in our analyses.

*Electron microscopy transmission (TEM)*

We investigated 22 ultra-thin cross-sections of feather barbules samples from 20 bird species with TEM. For each feather sample, a single barb with adjacent barbules was plucked from the feather rachis. We followed the protocol of Shawkey et al. (2003): The samples were washed with 0.1% Tween and 0.25M sodium hydroxide and then incubated for 30 minutes. They were then transferred to a 2:3 formic acid and ethanol solution and incubated for 2.5 hours. In the following step, ethanol was added to the samples in which they were incubated for 10 minutes. This step was repeated once. Propylene oxide was then added to the sample in which they were incubated for 10 minutes. All incubations were performed in glass vials on a bench shaker. We used Epon resin to infiltrate the samples with successive concentrations of 15%, 50%, 70% and 100%. Incubation of samples in each Epon concentration lasted for 30 hours. Samples were then placed in molds and left to polymerize in 24℃ for 48 hours.

Each resin block was then cut with RMC-MT ultramicrotome 6000. To obtain cross-sections of melanosomes, care was taken to have the cutting surface perpendicular to the barbule plane. The thickness of each section was between 70 and 100 nm. Cross-sections were placed on copper grids and observed with an FEI Morgagni 268D transmission electron microscope, operated at 70 kV.

Supplementary figures

**
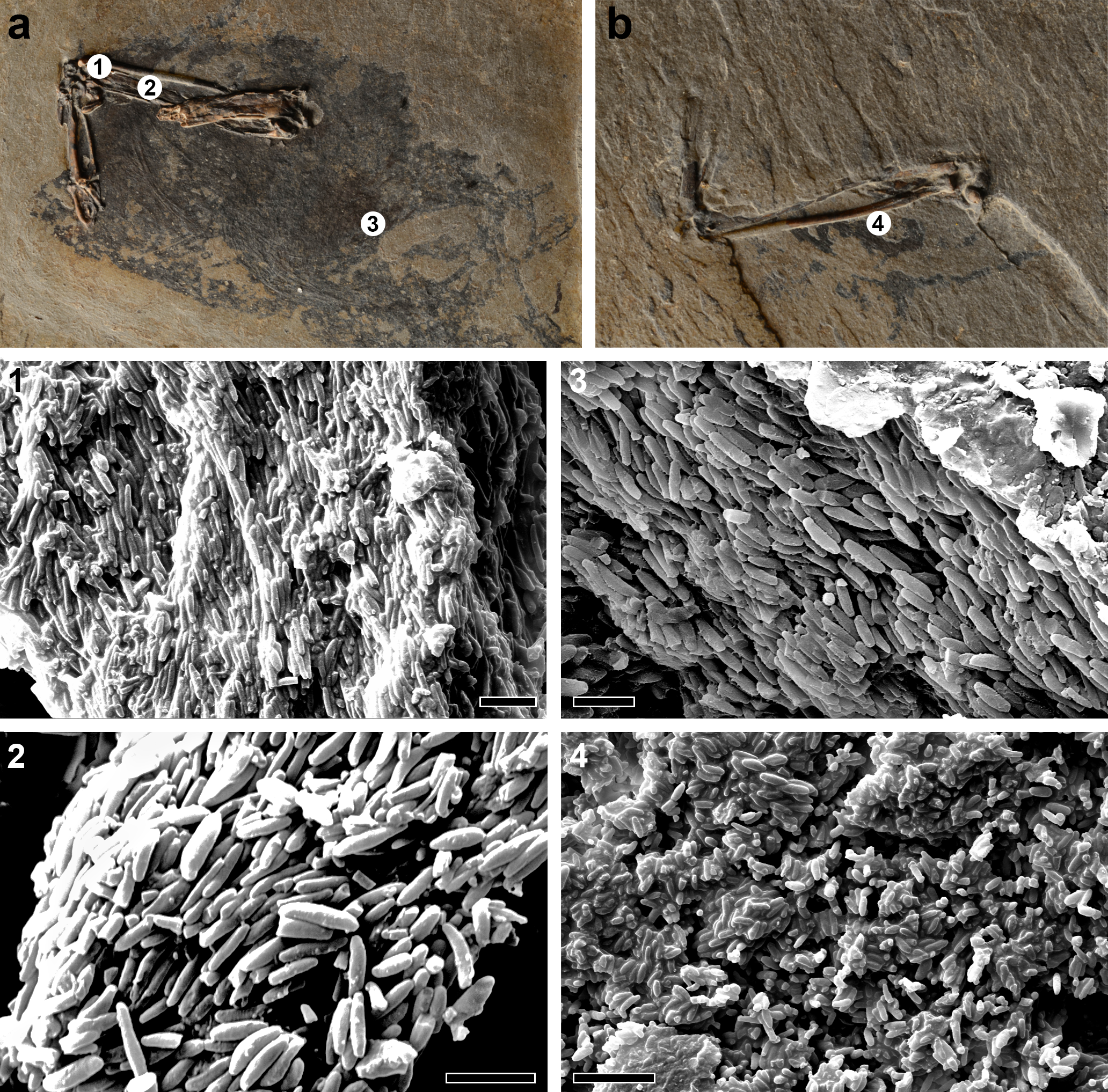
**

**Figure S1**. Sample sites and SEM images of samples for cf. *Primotrogon* sp. Samples are marked with white dots and numbered. a) *Primotrogon* sp. (SMF Av 498a). b) cf. *Primotrogon* sp. (SMF Av 498b). 1-4, corresponding SEM images to sample sites in a and b. Scale bars in 1-3 equal 2μm, scale bar in 4 equal 1μm.

**
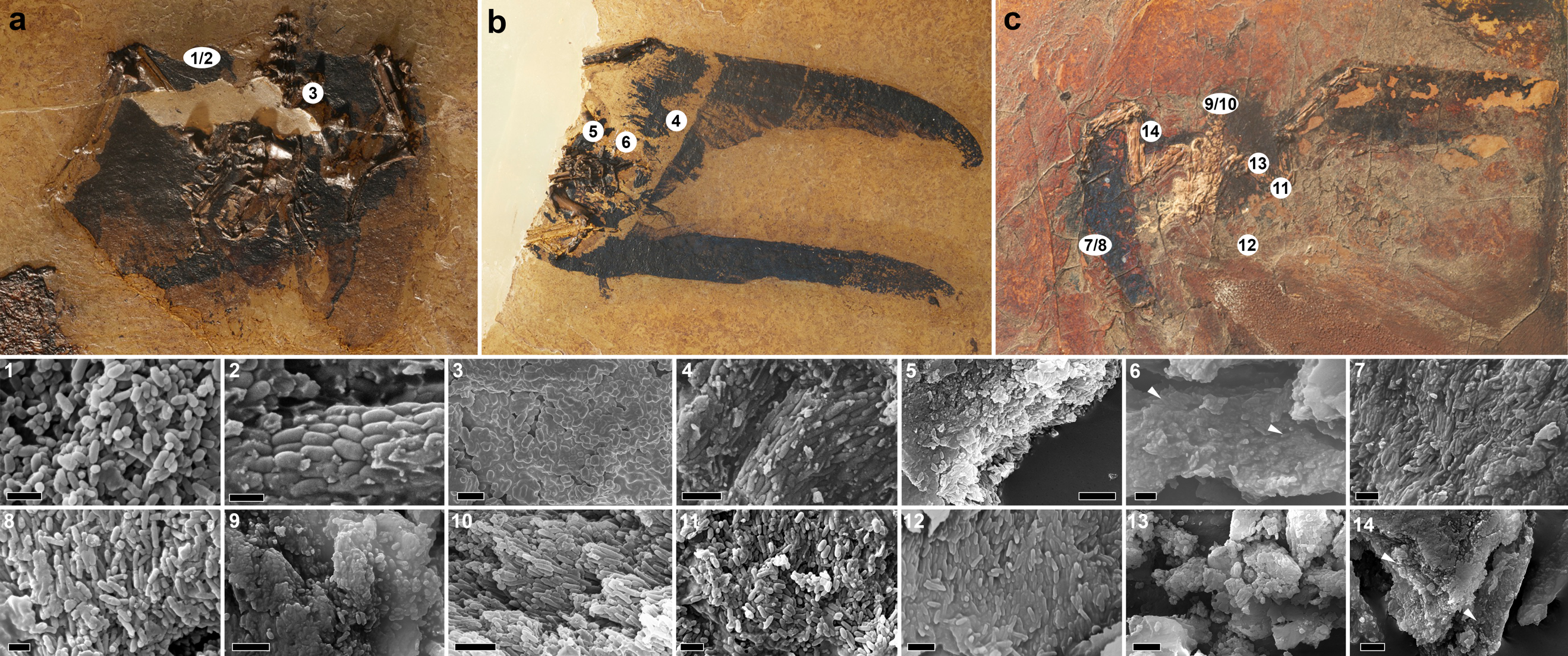
**

**Figure S2.** Sample sites for *S. szarskii.* a) *S. szarskii* (SMF-ME 11345B), b) *Scaniacypselus szarskii* (SMF-ME 11345A) c) *S. szarskii* (SMF-ME 599). Sample sites are marked with white dots and numbered. 1-14, corresponding SEM images to sample sites in a-c. Scale bars equal 1μm in 1-2, 4 and 8, 2μm in 3 and 10-12, 4μm in 5,9 and 13, and 5μm in 14. White arrows in panel 6 and 14 points to examples of melanosomes.


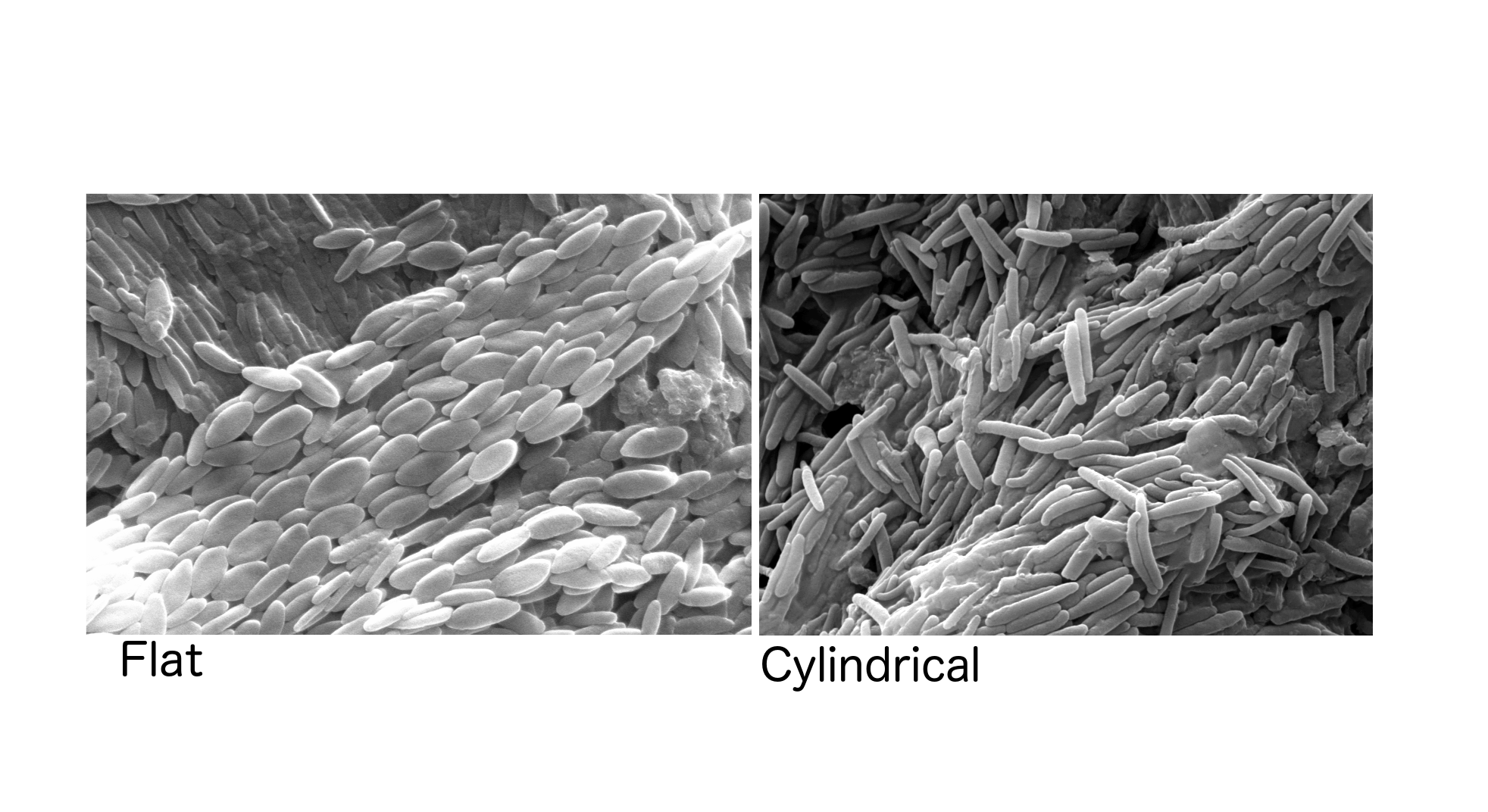


**Figure S3.** Flat/cylindrical morphology could easily be assessed using SEM images. Example of melanosome sample classified as exhibiting flat morphology (left) and a sample exhibiting cylindrical morphology (right).

**Figure S4.** Diameter and length for solid cylindrical melanosomes in the Li et al. (2012) data (black) and our data (white).

**Figure S5.** Sample size distribution in the Li et al. (2012) dataset (black) and our data set (white).

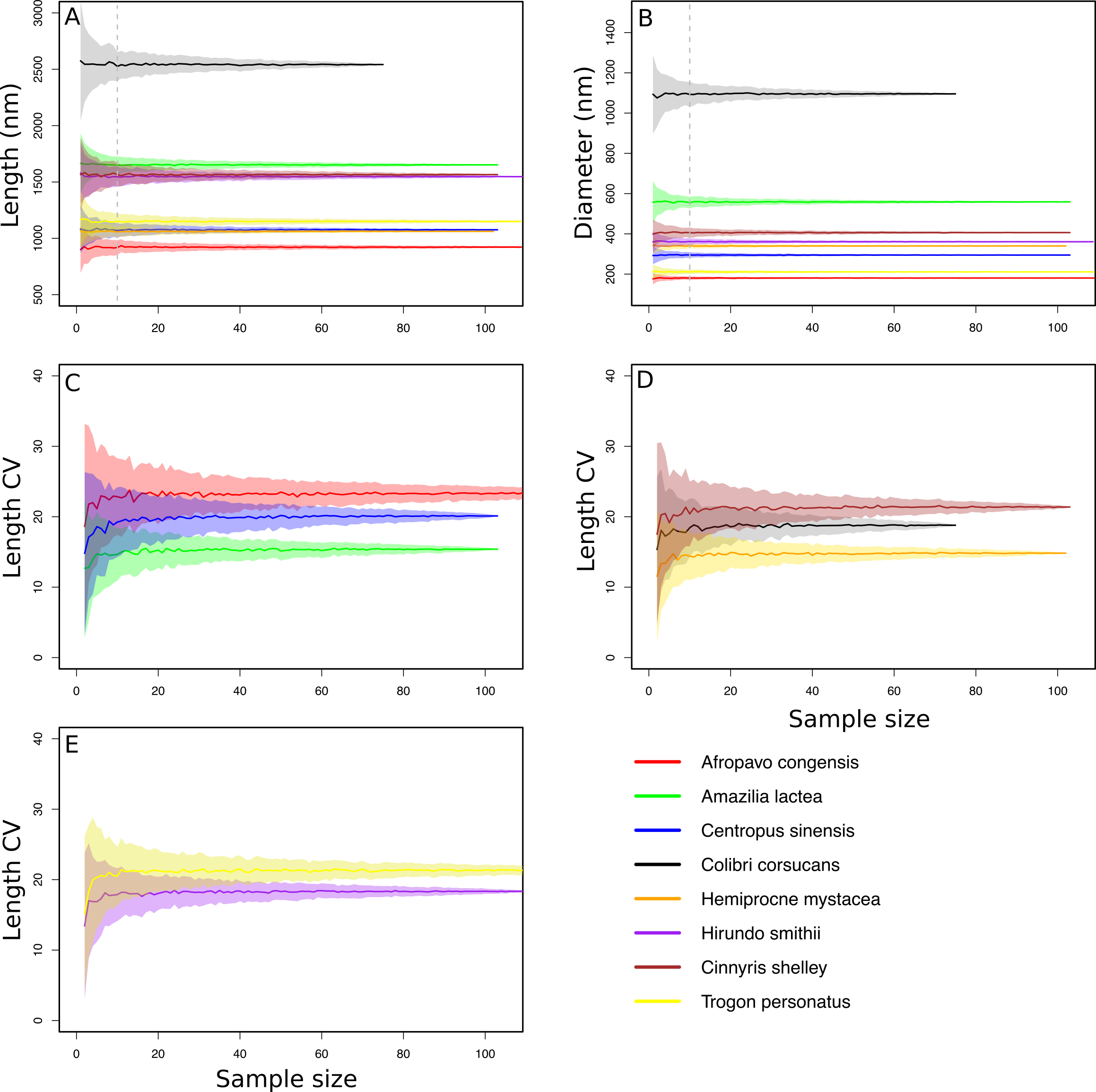


**Figure S6.** Effect of sample size on morphological variables. A sample of n, where n is 1-100, was drawn 200 times from the original distribution of eight species. The resulting distribution for increasing sample size is shown for length (A), diameter (B), and coefficient of variation of length (C-E). The solid line marks the mean of the 200 draws.


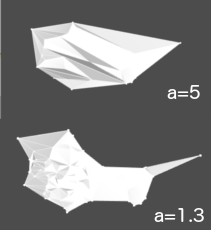


**Figure S7.** Example of the effect of changing the alpha parameter from a higher number (top) to a lower value (bottom). Areas with few data points have been “scooped out” resulting in a smaller volume for low alpha values.

**Figure S8.** Informal phylogeny used for constructing a phylomorphospace. Trimmed versions of this tree were used for calculating phylogenetic signal and convergence analysis.

**Figure S9.** Loading plot for PCAmix analysis. ld = aspect ratio.


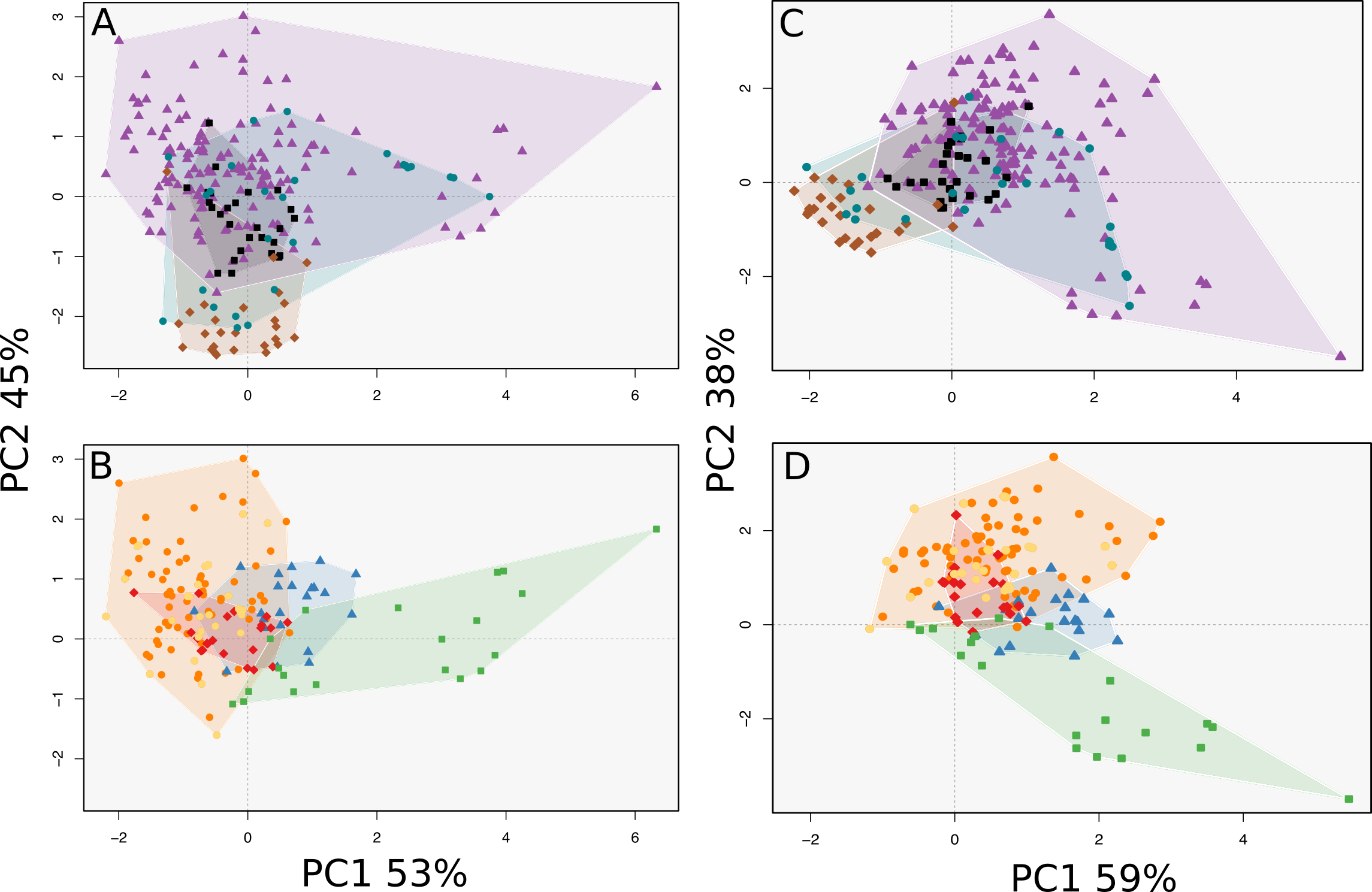


**Figure S10.** Melanosome morphospaces for PCA and pPCA analyses. Morphospace occupation for melanosomes from black, brown, grey and iridescent feathers using PCA scores (A) and pPCA scores (C). Morphospace occupation for iridescence-generating melanosomes subdivided in four categories (solid cylindrical/solid flat/hollow cylindrical/hollow flat) using PCA scores (B) and pPCA scores (D). Convex hulls are draw around each group (shaded areas), color code as in Fig. 2.


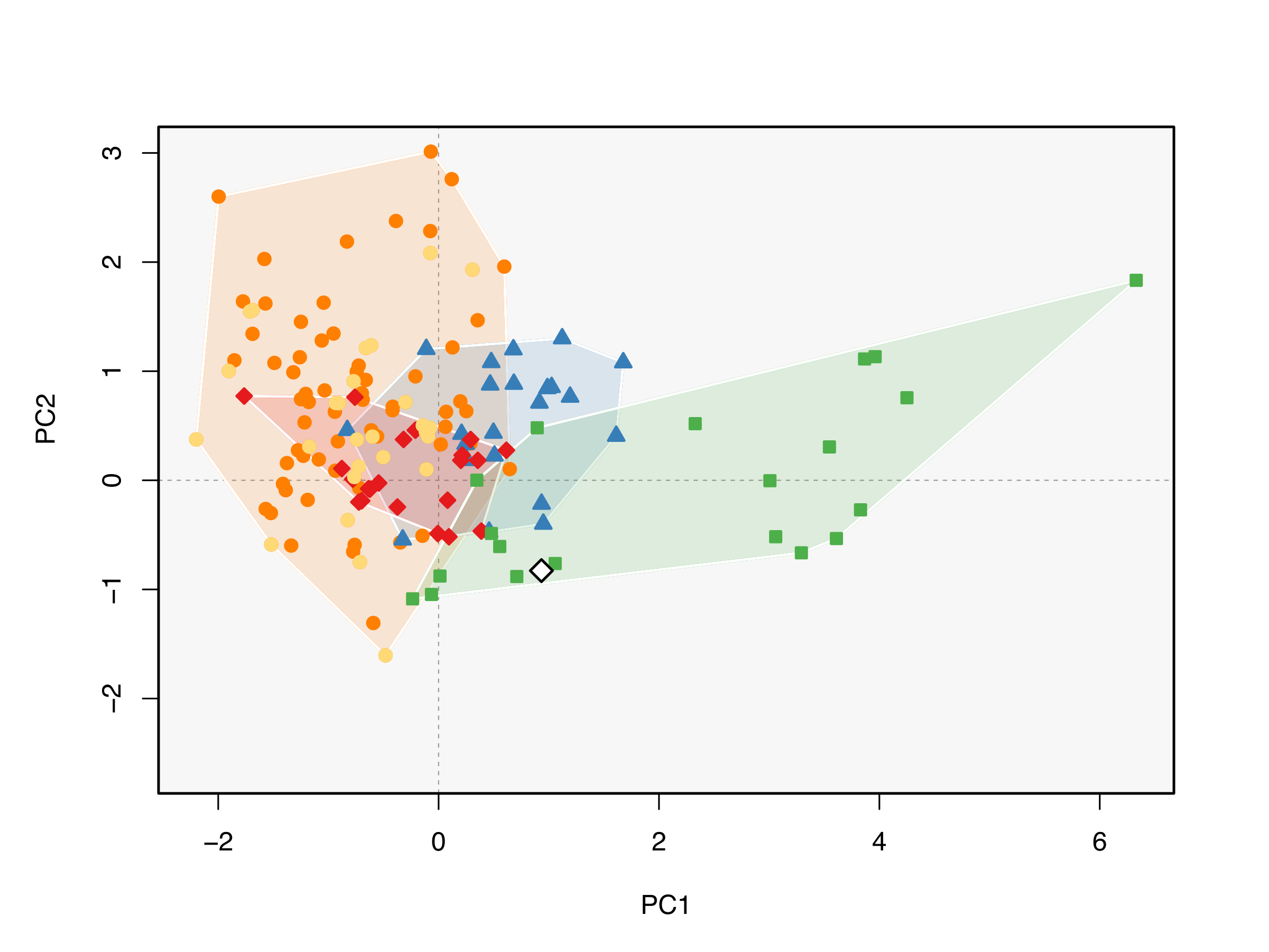


**Figure S11.** Morphospace position of flat melanosomes of the Cretaceous theropod *Caihong juji* (white diamond, after data given in Hu et al. 2018) in a PCA plot excluding flat/hollow dimensions. Color code as in Fig 2. Note that the length/width dimensions of *Caihong* are similar to that of modern birds with flat melanosome morphologies (blue and green points).


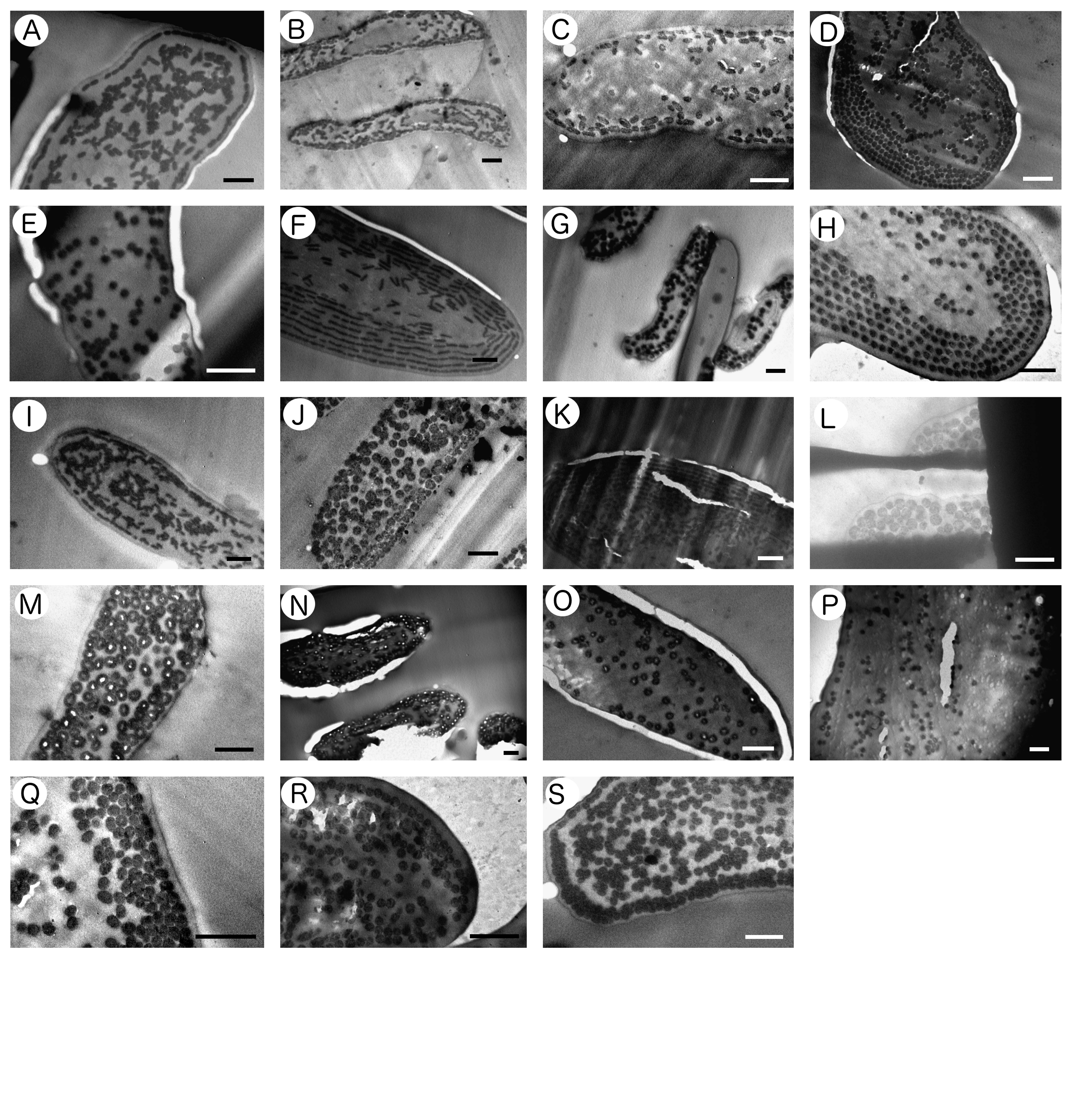


**Figure S12.** Cross sections of barbules of feather samples that were checked for melanosome hollowness. A) *Phaenicophaeus diardi diardi,* B) *Hirundo smithii,* C) *Tachycineta bicolor,* D) *Tauraco livingstonii*, E) *Psalidoprocne nitens,* F) *Chalcomitra senegalensis,* G) *Euphagus cyanocephalus,* H) *Galbula albirostris,* I) *Phaenicophaeus curvirostris*, J) *Lybius dubius*, K) *Psophia crepitans*, L) *Molothrus oryzivorus,* M) *Crotophaga major,* N) *Centropus sinensis,* O) *Centropus ateralbus,* P) *Centropus violaceus,* Q) *Eudynamys scolopacea,* R) *Galbula leucogastra,* S) *Surniculus lugubris.* All scale bars equal 1μm*.*
